# Obituary

**Published:** 1878-02

**Authors:** 


### OBITUARY.

At a meeting composed of the members of the Odontological Society, of New York, and its invited guests, held on January 15th, 1878, to pass resolutions relative to the death of Prof. Geo. T. Barker, of Philadelphia, Dr. Lord was on motion of Dr. Ambler made president.

Drs. Francis, Dwinelle, Perry, Ambler, and Bronson were appointed a committee, which on motion of Dr. Jarvis, was instructed to publish the resolutions and the minutes of the meeting in the dental journals, and to send a copy to the family of the deceased, and to the faculty of the Pennsylvania College of Dental Surgery.

E. A. BOGUE, Secretary.

The committee therefore in accordance with the above instructions, respectfully present the following:

Whereas: Learning with deep regret and sorrow of the death of our friend and professional brother, Professor Geo. T. Barker, of Philadelphia, in the prime of his manhood, and of his usefulness to the dental profession and to humanity, therefore,

Resolved, That we appreciate the upright, conscientious and earnest services of Prof. Barker, and remember with feelings of gratitude and admiration his eminent ability, his untiring research, and his candid and generous action in all matters, pertaining to the world of professional thought and investigation.

Resolved, That our regret for his untimely dmise is lightened by the remembrance that his services to dentistry will insure that his name and memory will always occupy an honorable and distinguished place among those of our profession, whom we all delight to honor.

Resolved, That our sympathies, true and heartfelt, are hereby tendered to the family, relatives and friends of the deceased, in this sad and inscrutable dispensation of divine providence.

Resolved, That we tender to the faculty of the Pennsylvania College of Dental Surgery an expression of the deep sense of the loss they have sustained in the death of him who has been so long associated with the institution and its interests.

Chas. E. Francis,
Wm. H. Dwinelle,
S. G. Perry,
J. G. Ambler,
Wm. A. Bronson,
Committee.

